# The Effect of Water Stress on Some Morphological, Physiological, and Biochemical Characteristics and Bud Success on Apple and Quince Rootstocks

**DOI:** 10.1155/2014/769732

**Published:** 2014-03-04

**Authors:** Ibrahim Bolat, Murat Dikilitas, Sezai Ercisli, Ali Ikinci, Tahsin Tonkaz

**Affiliations:** ^1^Department of Horticulture, Faculty of Agriculture, Harran University, Sanliurfa, Turkey; ^2^Department of Plant Protection, Faculty of Agriculture, Harran University, Sanliurfa, Turkey; ^3^Department of Horticulture, Faculty of Agriculture, Ataturk University, Erzurum, Turkey; ^4^Department of Biosystem Engineering, Faculty of Agriculture, Ordu University, Ordu, Turkey

## Abstract

The effects of different water stress (control, medium, and severe) on some morphological, physiological, and biochemical characteristics and bud success of M9 apple and MA quince rootstocks were determined. The results showed that water stress significantly affected most morphological, physiological, and biochemical characteristics as well as budding success on the both rootstocks. The increasing water stress decreased the relative shoot length, diameter, and plant total fresh and dry weights. Leaf relative water content and chlorophyll index decreased while electrolyte leakage increased with the increase of water stress in both rootstocks. An increase in water stress also resulted in reduction in budding success in Vista Bella/M9 (79.33% and 46.67%) and Santa Maria/MA (70.33% and 15.33%) combinations. However, the water stress in Santa Maria/MA was more prominent. The increase in water stress resulted in higher peroxidase activities as well as phenol contents in both rootstocks. Although catalase activity, anthocyanin, and proline contents increased with the impact of stress, this was not statistically significant. The results suggest that the impact of stress increased with the increase of water stress; therefore, growers should be careful when using M9 and MA rootstocks in both nursery and orchards where water scarcity is present.

## 1. Introduction

In semiarid and arid regions particularly during spring and summer months, the evaporative demand for the atmosphere results in significant drought stress in many crop plants, which is one of the most severe environmental stresses and affects almost all plant functions. In these conditions, water stress causes serious reduction in growth, quantity, and quality in many plants [1, 2]. It frequently occurs in both intensive fruit orchards and nurseries in many parts of the world. This situation directed researchers to make further investigations to reduce severe effects of water stress on different plant species. Therefore, new approaches including effective use of water, selection of drought resistant species, cultivars, and rootstocks have been considered to reduce the effects of water stress.

Severity of water stress has a great impact on the physiological and biochemical process of plants [3, 4]. Plant responses to water stress are usually screened on the level of selected physiological parameters such as water potential, relative water content, stomatal reactions, photosynthesis, or osmotic adjustment which have been proven to be good indicators of drought in several studies [3, 5, 6]. In addition, studies of carbohydrate accumulation and characterization of fruit trees to drought stress have also been involved [7] as well as plant hormones such as ethylene production [8, 9]. In recent studies, active oxygen species (AOS) and antioxidative enzymatic responses have also been proposed [10–13].

A nursery plant is composed of two parts; stock and scion. The rootstock has potential to affect the characteristics of scion (precocity, growth, productivity, fruit quality, and resistance to biotic and abiotic stress) [14]. The rootstock which has ability to tolerate partial or fully drought condition is the best material for budding because it has been stated that, in grafted plants, amount of water transporting from roots to shoots is controlled by rootstock [15, 16] and also drought tolerance of rootstock is conferred to grafted scions [15]. Tolerance levels of rootstocks which belong to different fruit species against environmental stress have been intensively studied recently [13, 17, 18]. The possible drought and irrigation problems would definitely affect the rootstock growth and the budding success, and, therefore, it would result in reduction in nursery and crop productivity as well as economic loss. However, there is limited information on grafting and budding success rate in rootstocks under water stress conditions.

More recently, the international well-known standard M9 and MA rootstocks have been used in intensive fruit orchards for apples and pears particularly in semiarid and arid conditions where water stress is the main issue. Therefore, the aim of the study was to identify budding efficiency and some physiological and biochemical responses of the rootstocks based on the parameters evaluated in dwarf apple and quince rootstocks.

## 2. Materials and Methods

### 2.1. Plant Material and Experimental Conditions

This study was carried out in Pome Fruit Research Center of Harran University, from April 2008 to April 2009. The climate of the region is classified as Mediterranean type with hot and extremely dry summers and wet winters, with average annual rainfall of 370 mm that mostly occurs during the autumn and winter months. July and August are the warmest months with almost no rainfall.

In this research, dwarf M9 (apple) and MA (quince) rootstocks were used and Vista Bella (apple) and Santa Maria (pear) cultivars were used as scions, respectively. Homogenously selected rootstocks from cutting propagated one-year old M9 apple and MA quince were planted into 10-litre volume of plastic pots filled with peat moss and sand mixture (3 : 1 v/v) in the beginning of April. The exposed top of growth medium within the pot was covered with an opaque plastic film during the experimental period and tied around the base of the stem to prevent vapor phase water movement within the growth medium and to minimize water loss from the pot surface. All plants were then placed on a bench in outside conditions. A polyethylene (PE) cover was also employed in case of rain during the experimental period.

### 2.2. Irrigation Treatments

Potted rootstock plants were equally irrigated until the start of the experiment (April till mid-July). The amount of water applied to each pot was determined by preliminary studies based on the pot capacity (PC). Ninety plants were divided into three groups for each rootstock to start water stress treatments consisting of control (100% of PC), moderate water stress (75% of PC), and severe water stress (50% of PC). Maintaining the moisture level regularly equivalent to 50% and 75% of PC imposed deficit irrigation. Control plants were maintained at full PC throughout the experiment. Each treatment was set up in a randomized block design with three replicates, each consisting of 10 plants. Water stress irrigation treatments were imposed from mid-July until the beginning of dormant period. Meanwhile, in the beginning of September, 5 plants from each replicate out of each treatment were randomly selected for some morphological, physiological, and biochemical evaluation.

The rest of the plants in each replicate were used for chip budding and preserved until the following season. During the dormant period no irrigation was made. In the following season, spring, the budded plants were water-stressed as in the previous year until the budding success evaluation was completed. Fertilization of rootstocks was routinely applied with half-strength of Hoagland solution once a week during the experimental period.

### 2.3. Plant Measurements

Terminal shoot lengths and diameters were measured both at the beginning of water stress irrigation treatment (mid-July) and in the beginning of September in which 7-week water stress was achieved. To determine relative shoot length (RSL) and shoot diameter (RSD) increments, the following formula was employed [(Final shoot length (diameter) − initial shoot length (diameter)/Final shoot length (diameter)] × 100. At the end of this period, whole plants of M9 and MA rootstocks were uprooted and cleaned with tap water and fresh and dry weights were determined. Dry weight was obtained by oven drying at 105°C for 24 h.

### 2.4. Physiological Measurements

Leaf relative water content (RWC), chlorophyll index (CI), and electrolyte leakage (EL) were determined on the rootstocks. RWC was determined form the upper fully expanded young leaves at noon (1:00 pm) according to Yamasaki and Dillenburg [19]. Leaf relative water content was calculated according to the equation:

(1) $$\begin{array}{c} \text{RWC}\;\left(\%\right)=\frac{\text{Fresh}\;\text{weight}-\text{Dry}\;\text{weight}}{\text{Saturated}\;\text{weight}-\text{Dry}\;\text{weight}}\times 100. \end{array}$$

CI was evaluated in the upper fully expanded young leaves with a Field Scout CM1000 chlorophyll meter. The Field Scout CM1000 chlorophyll meter estimates chlorophyll content based on ratios of the amount of ambient and reflected light at 700 and 840 nm. Measurements were made on a clear day between 12 and 14 pm.

EL was assessed as described by Lutts et al. [20] using nine-leaf discs for each treatment. Fully expanded young leaf samples were washed three times with deionized water to remove surface-adhered electrolytes. Leaf discs were placed in closed vials containing 10 mL of deionized water and incubated at 25°C on a rotary shaker for 24 h; subsequently, electrical conductivity of the solution (*L*_*t*_) was determined. Samples were then autoclaved at 120°C for 20 min and the last electrical conductivity (*L*_0_) was obtained after equilibration at 25°C. The electrolyte leakage was calculated as follows:

(2) $$\begin{array}{c} \text{Electrolyte}\;\text{leakage}\;\left(\%\right)=\left(\frac{L_{t}}{L_{0}}\right)\times 100. \end{array}$$

### 2.5. Assay of Stress-Related Biochemical Parameters

Young fully expanded leaf samples from the rootstocks were collected for biochemical measurements. One-gram sample of leaves was weighed and ground with an ice-cold pestle and mortar with 10 mL 50 mmol L^−1^ phosphate buffer (pH 7.0). The homogenates were then centrifuged at 10,000 g for 15 min at 4°C. The supernatant filtered through two layers of cheese-cloth was used for the determination of enzymatic activities as well as protein determination. The homogenized leaf tissues were stored at −80°C until being used for the biochemical analyses.

Catalase (CAT, EC. 1.11.1.6) activity was measured by following the decomposition of H_2_O_2_ at 240 nm with a UV spectrophotometer (Shimadzu UV-1700) as described by Havir and McHale [21]. The reaction mixture consisted of 0.1 mL enzyme extract, 2.8 mL phosphate buffer (pH 7.4, 0.1 mol L^−1^) containing 4 mM Na_2_EDTA. The biochemical reaction was started by adding 0.1 mL 0.01 mol L^−1^ H_2_O_2_ in the reaction system. Samples without H_2_O_2_ were used as blank. The activity of CAT was calculated by the differences obtained at OD_240_ values at a 30 sec interval for 2 min after the initial biochemical reaction. A change of 0.01 units per minute in absorbance was considered to be equal to one unit CAT activity. The activity of enzyme was expressed as U (unit) mg^−1^ protein.

The activity of POD (EC. 1.11.1.7) was assayed following Tuna et al. [22] with slight modifications. A 100 *μ*L of the plant extract was added to 3 mL of assay solution consisting of 3 mL of reaction mixture containing 13 mmol L^−1^ guaiacol, 5 mmol L^−1^ H_2_O_2,_ and 50 mmol L^−1^ Na-phosphate buffer (pH 6.5). An increase in absorbance at 470 nm for 3 min at 25°C was recorded on a UV-spectrophotometer (Shimadzu UV-1700). A change of 0.01 units per minute in absorbance was considered to be equal to one unit POD activity, which was expressed as U (unit) mg^−1^ protein.

Anthocyanin contents of leaves were determined according to the method of [23]. The leaf (1 g) sample was extracted in 3 mL methanol-HCl (1% HCl, v/v); the leaf samples were then left at 4°C in the fridge for 48 hours. Then the extract was filtered through 2 layers of cheesecloth and the anthocyanin content was measured by spectrophotometrically (Shimadzu UV-1700) at 530 and 657 nm wavelengths. The results were expressed as ΔA_(530–657)_ g^−1^ Fwt (fresh weight).

Phenol content of leaves was determined according to Singleton and Rossi [24] by using Folin-Ciocalteu solution with slight modifications. Fresh leaf tissues of 0.5 g were homogenized in liquid nitrogen, then extracted with 5 mL 80% methanol, and centrifuged at 10000 g for 10 min. A 100 *μ*L of the supernatant was mixed with 1.8 mL Folin-Ciocalteu solution (10 times diluted), shaken vigorously, and then kept 5 min at room temperature. Then 1.2 mL of 20% Na_2_CO_3_ was added and the volume made up to 6 mL with distilled water. The mixture was incubated for 1 h at 80°C and the absorbance was read at 765 nm against methanol. The results were expressed as mg gallic acid/g Fwt equivalent. The standard curve was prepared from a stock solution of gallic acid (4 mg/mL) in which the curve was prepared by dissolving various concentrations of gallic acid (0.1–1.8 mg/mL) in 80% methanol. The standard solution of gallic acid was reacted as stated above.

Proline content of the leaves was measured according to Bates et al. [25]. Proline was extracted from 0.5 g of leaf sample by grinding in 10 mL of 3% sulphosalicylic acid and the mixture was then centrifuged at 10000 g for 10 min. Two mL of the supernatant was then added into test tubes to which 2 mL of freshly prepared acid-ninhydrin solution and 2 mL of glacial acetic acid were mixed. The tubes were placed in a water bath for 1 h at 90°C and the reaction was terminated in ice-bath. The mixture was then extracted with 5 mL toluene and vortexed for 15 sec. After allowing standing at least for 20 min in darkness at room temperature to separate the toluene and aqueous phase, the toluene phase was then carefully collected into test tubes and the absorbance of the fraction was read at 520 nm with a Shimadzu UV-1700. The proline content in the sample was expressed as *μ*g g^−1^ fresh weight. The standard curve was prepared by employing L-proline.

Protein contents of the samples were determined according to Coomassie Brilliant Blue G250 method using bovine serum albumin as standard measured at 595 nm colorimetric wavelength [26].

### 2.6. Chip Budding

At the beginning of September (after 7-week water stress treatments), chip budding was made on the rest of the rootstocks. Vista Bella apple cultivar and Santa Maria pear cultivarbudded on M9 apple and MA quince, respectively. All rootstocks budded approximately 5 cm above the pot soil surface. After inserting the buds, they are taped with white rubber. The ties were removed off five weeks following budding to avoid damages resulting from the trunk expansion in the stock. During the dormant period, the stocks were headed back to 10 cm above the budding. Budding success was evaluated on the first week of coming April at which time shoot growth on successful buds was about 2.5 cm.

### 2.7. Statistical Analysis

The obtained data as % were converted to arc sin. Transformation and statistical analysis were made through these values. The data of the experiment were subjected to analysis of variance (ANOVA) using LSD test at the significance level of 0.05 or 0.01.

## 3. Results and Discussion

In this study, water stresses were evaluated on shoot growth parameters in two rootstocks. Water stress caused significant reductions in the increment of RSL in M9 and MA rootstocks (Table 1). Decline in RSL was more severe at “severe water stress” conditions in MA than that happened in M9 rootstocks. At the same conditions, decline in RSD was 36.63% in M9 and 40.69% in MA rootstocks. Significant differences were recorded for all watering regimes. Plant growth was also reduced under the negative effects of water stress in apple [27] and pear [17] depending on the rootstocks. The apple and quince rootstocks responded similarly to water stress on accumulation of fresh and dry weight (Table 1). Water stress inhibited fresh and dry weight accumulation in both rootstocks. Fresh weight decreased by 35–39% while dry weight decreased by 21–26% in both rootstocks at severe water stress conditions. Therefore, drought-induced decreases of rootstocks in terms of fresh and dry weights were accompanied with the increase of water stress. Similarly, Thomas Fernandez et al. [27] reported that the total plant dry weights of apple (Imperial Gala) were significantly reduced on M9 EMLA, MM.111, and Mark rootstocks, although Mark was the most sensitive of the tree rootstocks. Sakalauskaite et al. [28] reported 50% decrease in fresh and dry weights following five weeks of drought treatments in apple rootstocks.

RWC is considered as an important criterion of plant water status, so leaf RWC was found as 70.20, 77.82, and 81.65% in M9 and 63.80, 72.80, and 79.85% in MA for the severe, moderate, and control water stress irrigation regimes, respectively (Table 2). In two rootstocks, RWC decreased with the increasing levels of water stress. Similarly, it is reported that in pear [17] and in *Malus prunifolia* and *Malus hupehensis* [13], the negative effects of water stress on leaf RWC were reduced depending on the rootstock genotypes. Leaf RWC reflecting the metabolic activity in tissues [29] declined significantly due to water stress (Table 1). Such a decrease in leaf RWC could have been due to unavailability of water in the soil [30], or root systems, which are not able to compensate for water, lost by transpiration through a reduction of absorbing surface [31].

Water stress regimes also resulted in decrease in CI levels in both rootstocks during the period of stress (Table 2). Leaf CI in severe water stress case was reduced by 46% in M9 and 51% in MA rootstocks compared to the control. Similar findings were also reported by Alizadeh et al. [18] on apple rootstocks, by Haifeng et al. [11] on citrus rootstocks, and by Gholami et al. [12] on figs.

Deficit irrigation also reduced EL on M9 compared to MA rootstocks (Table 2). Humidity contents were reduced in both pots of rootstocks and, as a result of that, EL content increased. For example, EL level increased from 25.32% to 30.95% in M9 and from 39.06% to 52.17% in MA rootstocks. Similarly, increase of EL was determined by Gholami et al. [12] in figs. EL increase is accompanied with the increase of cell permeability; thus, an important strategy for the development of drought resistance should involve the maintenance of cell membrane integrity. For example, Wang et al. [13] compared two apple rootstocks: *Malus prunifolia* (drought tolerant) and *Malus hupehensis* (drought sensitive) for water stress. Irrigation withheld for 12 d led to considerable ultrastructural alterations in organelles in which *M. prunifolia* maintained their structural cell integrity longer than did *M. hupehensis*.

**Figure 1:**
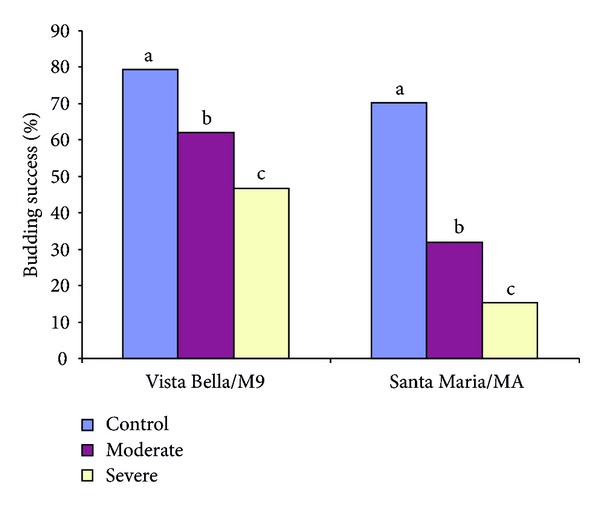
Budding success of Vista Bella/M9 and Santa Maria/MA grown under before and after budding water stress conditions. (Different letters shown in bars in each rootstock show the significant differences in 1% LSD level.)

Water stress negatively affected the success of budding in both rootstocks. Increase in the water stress resulted in decreasing of budding success. The budding success was 79.33% in Vista Bella/M9 combination in control group while it was 46.67% in severe water stress treatment (Figure 1). In the case for Santa Maria/MA combination, budding success dropped from 70.33% to 15.33% (Figure 1). Similar situation was reported by Sauve et al. [32] for Golden Delicious/M111 budding combination, which was severely affected before and after chip budding in −5 to −25 kPa water stress applications. Since above treatments reflected the condition of semiarid and arid regions, budding efficiency is, therefore, significantly affected. In our study, budding success was about 70 and 80% in MA and M9 rootstocks in control group. However, this rate significantly dropped with the increase of water stress. It is important to note that the budding success ratio in drought conditions is higher within the Vista Bella/M9 than Santa Maria/MA. It is thought that water uptake and translocation during the budding are more efficient within the genus than between different genera.

There were also strong correlations between vegetative parameters, biomass production, CI, EL, and budding success for each rootstock (Table 3). In addition, morphological, physiological, and biochemical parameters of apple and quince rootstocks to water stress were quite variable. This variability could be associated with the rootstock, water stress level, intensity of stress, and environmental conditions.

**Figure 2:**
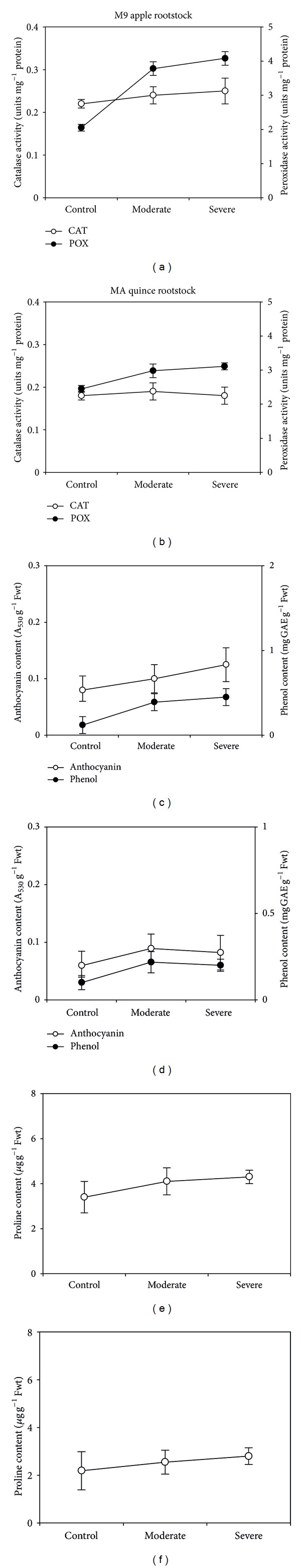
Biochemical parameters obtained from the rootstocks (M9 and MA) grown in various water stress levels. ((a)-(b)) Catalase and peroxidase enzymes; ((c)-(d)) anthocyanin and phenol; ((e)-(f)) proline contents in both rootstocks. (Vertical bars are standard deviation (SD) of means.)

When biochemical parameters were evaluated, catalase level did not show significant difference from the control group although peroxidase activity in both rootstocks was higher compared with plants under control conditions (Figures 2(a) and 2(b)). A similar phenomenon was also observed by Gholami et al. [12] who found that peroxidase levels were higher in drought affected fig plants than in control plants although no statistical difference was obtained. They also reported that catalase levels did not show great difference between control and drought affected plants. Similar findings were also reported by Wang et al. [13] who suggested that oxidative damage was minimized with the increase of enzymatic and metabolic responses. Again, anthocyanin and phenol contents in water-stressed plants of both rootstocks were higher than the control but only phenol contents were found significantly different from control plants (Figures 2(b) and 2(c)). A similar trend was also shown for proline contents. Although much attention was given to biochemical parameters in drought stressed plants such as vegetables and trees, the results obtained from vegetables in short term had great impact to evaluate stress mechanisms. In trees, due to long-term stress period, it is difficult to see the differences between treatments if any significant differences had occurred. Instead, it would be more convenient to examine the histological structures of plant vessel elements before and after water stress where budding was made. However, biochemical parameters in this study showed that M9 apple rootstock performed better than MA quince rootstock when the biochemical parameters were considered. It should be also remembered that the adaptation period of trees to water stress might have prevented us from measuring the differences between rootstocks; however, the budding success was found related to the activities of the tested enzymes, proline, and phenol metabolism which varied from one stress level to another although a difference was not found, possibly due to the above-mentioned reasons. According to these studies, to achieve a high level of budding success, plant materials should not be water-stressed during budding period.

## 4. Conclusions

In view of findings of the present study, it can be concluded that water stress strongly affected both rootstocks and decreased plant growth of M9 and MA rootstocks. The water stress resulted in reduction in growth parameters by decreasing relative shoot length and diameter and plant total fresh and dry weights. Leaf relative water content and chlorophyll index decreased while electrolyte leakage increased with the increase of water stress in both rootstocks. The increase in water stress resulted in higher peroxidase activities as well as phenol contents in both rootstocks. Although catalase activity, anthocyanin, and proline contents increased with the impact of stress, this was not significant. The effect of severe water stress conditions was more visible between genera grafting (Santa Maria pear/MA quince rootstock) than within genera (Vista Bella apple/M9 apple rootstocks). This should be taken into account where water stress is a limiting factor in M9 and MA nursery and orchards. A further detailed study is also needed to elucidate the underlying biochemical processes and anatomical and genetic parameters which are responsible for differential responses of apple and pear rootstocks and also grafting combinations.

## Figures and Tables

**Table 1:** The effects of water stress on M9 apple and MA quince rootstocks increment of relative shoot length (RSL), and relative shoot diameter (RSD), and plant total fresh and dry weights.

Water stress	RSL increment (%)	RSD increment (%)	Plant fresh weight (g)	Plant dry weight (g)
M9				
Control	8.21^a^	5.76	171.44^a^	75.15^a^
Moderate	4.63^a^	5.27	137.82^b^	67.66^b^
Severe	2.65^b^	3.65	112.39^c^	59.56^c^
LSD	3.92*	n.s.	12.26**	5.02*

MA				
Control	25.01^a^	4.40	187.25^a^	82.19^a^
Moderate	22.97^a^	4.01	143.38^b^	70.20^b^
Severe	5.09^b^	2.61	115.72^c^	61.19^c^
LSD	14.57**	n.s.	14.58**	4.79*

*F* test:*P < 0.05; ** <0.01; n.s.: not significant.

The means followed by the same letter in each column are not significantly different according to LSD test at 5% or 1% level.

**Table 2:** Relative water content (RWC), chlorophyll index (CI), and electrolyte leakage (EL) of M9 and MA rootstocks grown under water stress conditions.

Water stress	RWC (%)	CI	EL (%)
M9			
Control	81.65^a^	270.33^a^	25.32^b^
Moderate	77.82^a^	228.70^b^	27.27^b^
Severe	70.20^b^	143.66^c^	30.95^a^
LSD	7.52*	16.38**	2.16*

MA			
Control	79.85^a^	271.00^a^	39.06^b^
Moderate	68.80^b^	208.60^b^	44.30^b^
Severe	60.80^c^	132.50^c^	52.17^a^
LSD	6.49*	14.75**	4.20**

*F* test: *P < 0.05; ** <0.01; n.s.: not significant.

The means followed by the same letter in each column are not significantly different according to LSD test at 5% or 1% level.

**Table 3:** Relationships between some parameters in M9 apple and MA quince rootstock.

	WS	RWC	RSL increment	RSD increment	Plant fresh weight	Plant dry weight	CI	EL	Budding success
M9									
WS	1.00**	0.90**	0.78*	0.75*	0.89**	0.81**	0.88**	−0.88**	0.94**
RWC		1.00**	0.74*	0.81**	0.86**	0.82**	0.89**	−0.91**	0.92**

MA									
WS	1.00**	0.91**	0.71*	0.75*	0.87**	0.84**	0.90**	−0.89**	0.87**
RWC		1.00**	0.57 ^n.s^	0.82**	0.82**	0.85**	0.91**	−0.88**	0.85**

Units: water stress (WS), relative water content (RCW), relative shoot length (RSL), relative shoot diameter (RSD), chlorophyll index (CI), and electrolyte leakage (EL); n.s.: not significant; *P* < 0.05*;  *P* < 0.01**.
